# Investigating Roasted Açaí (*Euterpe oleracea*) Seed Powder as a Coffee Substitute: Effects of Water Temperature, Milk Addition, and In Vitro Digestion on Phenolic Content and Antioxidant Capacity

**DOI:** 10.3390/foods14152696

**Published:** 2025-07-31

**Authors:** Rayssa Cruz Lima, Carini Aparecida Lelis, Jelmir Craveiro de Andrade, Carlos Adam Conte-Junior

**Affiliations:** 1 Technological Development Support Laboratory (LADETEC), Center for Food Analysis (NAL), Federal University of Rio de Janeiro (UFRJ), Cidade Universitária, Rio de Janeiro 21941-598, RJ, Brazil; lima_rayssa@id.uff.br (R.C.L.); jelmirandrade@outlook.com (J.C.d.A.); conte@iq.ufrj.br (C.A.C.-J.); 2 Graduate Program in Veterinary Hygiene (PPGHV), Faculty of Veterinary Medicine, Fluminense Federal University (UFF), Vital Brazil Filho, Niterói 24230-340, RJ, Brazil; 3 Nanotechnology Network, Carlos Chagas Filho Research Support Foundation of the State of Rio de Janeiro (FAPERJ), Rio de Janeiro 20020-000, RJ, Brazil

**Keywords:** sustainability, seed extract, green extraction, infusion beverage, Amazonian fruit, bioactive compounds

## Abstract

Açaí (*Euterpe oleracea*) seeds account for up to 95% of the fruit’s weight and are commonly discarded during pulp processing. Roasted açaí seed extract (RASE) has recently emerged as a caffeine-free coffee substitute, although its composition and functionality remain underexplored. This study characterized commercial açaí seed powder and evaluated the effect of temperature on the recovery of total phenolic content (TPC) in the aqueous extract using a Central Composite Rotatable Design (CCRD). An intermediate extraction condition (6.0 ± 0.5 g 100 mL^−1^ at 100 °C) was selected, resulting in 21.78 mg GAE/g TPC, 36.23 mg QE/g total flavonoids, and notable antioxidant capacity (FRAP: 183.33 µmol TE/g; DPPH: 23.06 mg TE/g; ABTS: 51.63 mg TE/g; ORAC: 31.46 µmol TE/g). Proton Nuclear Magnetic Resonance (^1^H NMR) analysis suggested the presence of amino acids, carbohydrates, and organic acids. During in vitro digestion, TPC decreased from 54.31 to 17.48 mg GAE 100 mL^−1^ when RASE was combined with goat milk. However, higher bioaccessibility was observed with skimmed (33%) and semi-skimmed (35%) cow milk. These findings highlight RASE as a phenolic-rich, antioxidant beverage with functional stability when prepared with boiling water. This is the first study to report the phytochemical profile of RASE and its interactions with different milk types, supporting its potential as a coffee alternative.

## 1. Introduction

The promotion of healthier eating habits has increasingly become a priority, driven by the beneficial properties that certain foods can offer to human health, such as antioxidant potential. Among these, açaí (*Euterpe oleracea*) stands out as a native fruit of the Amazon Basin, primarily produced in northern Brazil [1]. Historically consumed by Indigenous communities, açaí has become a traditional food among the Amazonian population [2], and its popularity has expanded both nationally and internationally, including markets in the United States, Japan, China, and countries within the European Union [3]. In 2023, açaí production reached approximately 2 million tons, contributing over 8 billion Brazilian Reais (R$) to the national economy [4].

Açaí is a globular fruit composed of three main parts: seed, mesocarp and epicarp. The seed, yellowish-brown in color, represents approximately 70 to 95% of the entire fruit. The mesocarp, located just above the seed, consists of a thin layer (1–2 mm), while the epicarp is the outermost and rigid layer, with the ripe fruit presenting different colors, green (“white açaí”) or purple (“dark açaí”) [3]. The açaí pulp is the most valuable component due to its nutritional content and the presence of bioactive compounds [5,6]. However, the edible portion constitutes only 7 to 25% of the entire fruit [2,7], with the seed being largely discarded. It is estimated that over 1,000,000 metric tons of seeds are improperly disposed of in the Amazon region, leading to significant environmental concerns [8,9].

Agro-industrial residues such as açaí seeds have attracted scientific interest for their content of bioactive compounds with potential health benefits [10], including application in the pharmacological field [11]. Extracts obtained from açaí seeds using various solvents and extraction techniques have revealed the presence of phenolic acids, flavonoids (particularly proanthocyanidins), stilbenes, and lignans [11,12], compounds associated with antioxidant [12,13,14], antimicrobial [12], anticancer [13], anti-inflammatory, and oxidative stress-preventing effects [15,16].

Recently, the use of roasted açaí seeds in powdered form to prepare an infusion beverage popularly known as “açaí coffee”, has emerged as a sustainable alternative [17]. This beverage, reproduces several sensory characteristics of coffee, such as aroma, flavor and perceived energizing effect [18], while serving as a caffeine-free option for individuals sensitive to caffeine. High caffeine intake has been associated with adverse effects such as increased blood pressure, hypercholesterolemia, gastrointestinal disorders, and functional dyspepsias [19,20]. Roasted açaí seed extract (RASE) could be an alternative as a functional beverage, and may offer health benefits attributed to the presence of bioactive compounds like anthocyanins with antioxidant activity [9,21]. Additionally, traditional coffee is often consumed with milk to reduce the bitterness and astringency of the extract [22]. Furthermore, little is known about how homemade preparation methods, such as using boiling water, affect the extraction and bioaccessibility of these bioactive compounds. In addition, milk components such as proteins, polysaccharides, and lipids can interact with polyphenols from various extracts, potentially influencing their release and absorption during in vitro simulated digestion [23]. Given these interactions, in vitro digestion models are widely applied to simulate gastrointestinal conditions and assess how milk addition may influence the release and bioaccessibility of polyphenols, particularly from emerging coffee substitutes such as RASE. Understanding these factors is essential to assess the health benefits that this novel beverage may offer to consumers.

Despite the increasing consumption of roasted açaí seed as an infusion beverage in some regions of Brazil, scientific data on its chemical composition, antioxidant potential, and behavior under digestion conditions are still lacking. To our knowledge, this is the first study to systematically investigate a beverage derived from roasted açaí seeds, a product traditionally prepared and consumed on a small scale, yet never explored in detail by the scientific community. No previous research has comprehensively evaluated the phytochemical profile, antioxidant activity, or polyphenol bioaccessibility of this infusion, particularly in combination with milk, a common consumer practice.

Filling this knowledge gap is essential not only to support the scientific understanding of this underutilized Amazonian by-product but also to provide the food industry and consumers with foundational evidence of its functional potential. In this context, our study assessed the effects of extraction parameters (specifically water temperature and powder concentration) on total phenolic compound (TPC) recovery using a Central Composite Rotatable Design (CCRD). We also characterized the phytochemical profile and major metabolites of the RASE through ^1^H Nuclear Magnetic Resonance (NMR) spectroscopy and evaluated its antioxidant capacity using four complementary assays: FRAP, DPPH, ABTS, and ORAC. Finally, and for the first time, we investigated the in vitro digestion and polyphenol bioaccessibility of RASE when combined with different types of milk (whole, semi-skimmed, and skimmed bovine milk, and whole goat milk), yielding novel insights into the nutritional and functional properties of this sustainable beverage.

## 2. Material and Methods

### 2.1. Chemical and Reagents

Chemical reagents for standard curves such as Trolox and gallic acid were purchased from Sigma-Aldrich (St. Louis, MO, USA). Sodium carbonate (Na_2_CO_3_), hydrochloric acid (HCl), methanol, sodium phosphate monobasic and dibasic (NaH_2_PO_4_ and Na_2_H_2_PO_4_), sodium chloride (NaCl), sodium hydroxide (NaOH), acetic acid (CH_3_COOH) was purchased from Synth (Diadema, São Paulo, Brazil). 2,4,6-Tris (2-pyridyl)-s-triazine (TPTZ), ferric chloride (FeCl_3_), 2,2-diphenyl-1-picrylhydrazyl (DPPH), 2,2′-Azino-bis (3-ethylbenzothiazoline-6-sulfonic acid) diammonium salt (ABTS), potassium persulfate, fluorescein, 2,2′-Azobis (2-methylpropionamidine) dihydrochloride (AAPH), pepsin, pancreatin, calcium chloride dehydrate (CaCl_2_ 2H_2_O), Folin–Ciocalteu, and ethanol was purchased from Sigma-Aldrich (St. Louis, MO, USA).

### 2.2. Experimental Design

A Central Composite Rotatable Design (CCRD) was employed to evaluate the effects of water temperature (T, °C) and proportion (Prop, g 100 mL^−1^) on the extraction of total phenolic compounds from RASE. The GAMMA-GUI software version 2.0, developed by the “Grupo de Análise Multivariada em Matrizes Alimentares” and based on the Matlab 2023a^®^ platform (The MathWorks Inc., Natick, MA, USA), was used for data analysis. This software is freely available for download on GitHub (https://github.com/appGAMMA (accessed on 10 June 2024)). The experimental design included two factorial levels (−1 and +1), a central point (0), and axial points (−1.4142 and +1.4142), as shown in Table 1. The experiment generated a matrix with 11 trials, including three replicates at the central point, conducted in random order. Total phenolic content (TPC) was used as the response variable.

The significance of individual effects was assessed by ANOVA at a 95% confidence level, fitting the experimental models to the observed responses. The overall fit of the model was evaluated by the coefficient of determination (R^2^), the adjusted coefficient of determination (R^2^ adjusted), ANOVA, and the analysis of the partition of the residual sum of squares into pure error and lack of fit. Contour plots were generated for the validated models, keeping the third independent variable constant at the central point. The sequential simplex algorithm was applied to optimize the experimental conditions, targeting in the highest TPC values. A second-order polynomial expression was fitted for each dependent variable (response), as shown in Equation (1):

(1) $$Y\;=\;b_{0}+\;b_{1}x_{1}+\;b_{2}x_{2}+\;b_{11}\;x_{1}^{2}+\;b_{22}x_{2}^{2}+\;b_{12}x_{1}x_{2}$$

where $b_{0}$ is the intercept; $b_{1}$ and $b_{2}$ are the regression coefficients for the linear effects; $b_{11}$ and $b_{22}$ are the regression coefficients for the quadratic effects; $b_{12}$ is the regression coefficient for the interaction effects; and $x_{1}$ and $x_{2}$ are the coded values for the independent variables.

### 2.3. Roasted Açaí Seed Powder and Extracts Samples

Given our objective to characterize a product currently available on the market, we selected a commercially distributed roasted açaí seed powder, obtained from the company Raízes do Açaí (Afuá, Pará, Brazil). According to da Silva Magalhães (2024) [24], the seeds are roasted by the company at 140 °C for 60 min and subsequently ground. The extract was prepared following the conditions described in Table 1. Immediately afterward, the extract was filtered using Melitta^®^ coffee filter nº 103 (Minden, Germany). After filtration, the extracts were protected from light and immediately refrigerated (MetalFrio^®^-São Paulo, Brazil) at −20 °C. To evaluate the extracts defined by the CCRD (Table 1), all extractions were performed in triplicate. Following the CCRD analysis, an additional extraction was conducted to recover the TPC from the aqueous extract in duplicate using the mid-range proportion used for CCRD (6 g per 100 mL of water at 100 °C), as no optimum point was identified (see Section 3.1). All extracts were analyzed on the same day as extraction.

### 2.4. Roasted Açaí Seed Extract Preparation to ^1^H NMR Analysis

The ^1^H NMR technique was employed to investigate the major compounds present in the polar extract of roasted açaí seeds. For this purpose, an extract was prepared by subjecting 0.12 g of the sample to 2 mL of deuterated water (deuterium oxide) at 100 °C. Due to the high cost of deuterated water, it was necessary to proportionally reduce the sample volume while maintaining the same extraction ratio established in Section 2.3 under the optimized CCRD conditions (6 g per 100 mL of water at 100 °C), solely to obtain sufficient material for NMR analysis.

Following the extraction, Nuclear Magnetic Resonance (NMR) experiments were conducted using a low-field Spinsolve Multi X spectrometer operating at a frequency of 60 MHz, equipped with a compact permanent magnet based on the Halbach design (Magritek Spinsolve, Wellington, New Zealand) [25]. This spectrometer features a gradient coil along the transverse plane of the NMR tube, capable of producing a maximum field gradient of 0.16 T m^−1^. It also includes an external lock system that enables the use of non-deuterated solvents, combined with an effective strategy for strongly attenuating solvent signals by employing highly selective solvent suppression methods, specifically the PRESAT method.

All NMR spectra were recorded at a temperature of 26.5 °C. The Spinsolve software version 2.3.8 was used for data acquisition, with standard values of 4.8 ppm and 2.5 kHz applied to the center and width of all spectra, respectively. The number of scans was 132, with two transients acquired per sample, each with a recycling delay of 3.8 s and 8k time-domain points sampled over an acquisition time of 3.2 s.

### 2.5. Phytochemical Characterization of the Roasted Açaí Seed Extract

The characterization was conducted in two sequential stages. In the first stage, each extract obtained under different conditions defined by the Central Composite Rotatable Design (CCRD) was analyzed for total phenolic content (TPC). In the second stage, the extract obtained under the optimized conditions was subjected to a comprehensive analysis. This included the evaluation of TPC before and after in vitro simulated digestion, determination of Total Flavonoid Content (TFC), assessment of antioxidant capacity using FRAP, DPPH, ABTS, and ORAC assays, and structural characterization by ^1^H NMR spectroscopy.

#### 2.5.1. Total Phenolic Content (TPC)

TPC was determined using the Folin–Ciocalteu reagent according to Lima et al. [26], with minor modifications. Each extract was previously diluted as required. After dilution, an aliquot of 100 µL of the extract was added to 500 µL of Folin–Ciocalteu 1:10 (*v*/*v*), homogenized, and kept at rest for 5 min in the absence of light. After that, 1500 µL of sodium carbonate 7.5% (*w*/*v*) sodium carbonate sodium and 1000 µL of ultrapure water were added, mixed, and left to stand for 2 h, at room temperature and in the dark. The absorbance was measured at 760 nm using the spectrophotometer UV–VIS (UV-1900i, Shimadzu, Kyoto, Japan). The calibration curve (75 to 600 mg L^−1^, with the equation of the line “y = 0.0024x + 0.069” and R^2^ = 0.9953) was calculated using gallic acid as standard.

#### 2.5.2. Total Flavonoid Content (TFC)

TFC was determined according to the method described by Lima et al. [26]. Prior to analysis, the extract was diluted 10 fold. Then, 500 µL of the diluted extract was mixed with 2 mL of ultrapure water and 150 µL of 5% (*w*/*v*) sodium nitrite solution. The mixture was allowed to stand for 5 min at room temperature. Subsequently, 150 µL of 10% (*w*/*v*) aluminum nitrate solution was added, and the mixture was allowed to react for 1 min at room temperature. Finally, 1000 µL of 1 mol L^−1^ sodium hydroxide and 1200 µL of ultrapure water were added, and the solution was kept at room temperature for 30 min. The absorbance was then measured at 425 nm using a UV–VIS spectrophotometer (UV-1900i, Shimadzu, Kyoto, Japan). The calibration curve (100 to 500 mg L^−1^), constructed using quercetin as the standard, followed the equation y = 0.001x − 0.0017, and R^2^ = 0.9991.

#### 2.5.3. Ferric-Reducing Antioxidant Power (FRAP)

FRAP was determined according to the method used by Lima et al. [26]. The antioxidant capacity was assessed in triplicate for each duplicate extract. Each extract was diluted 50 fold, after dilution an aliquot of 45 µL of the extract was added to 135 µL of ultrapure water and 1.35 mL of FRAP reagent (0.3 M acetate solution, pH 3.6; 10 mM TPTZ solution in 40 mM HCl; and 20 mM ferric chloride solution). Followed by homogenization for 10 s by vortex and subjected to a water bath (Thermo Scientific, Waltham, MA, USA) at 37 °C for 30 min, without light. The absorbance was measured at 595 nm using the spectrophotometer UV–VIS (UV-1900i, Shimadzu, Kyoto, Japan). The calibration curve (160 to 800 µM L^−1^, with equation of the line “y = 0.0012x + 0.0041” and R^2^ = 0.9955) was calculated using Trolox as standard, and was conducted in triplicate.

#### 2.5.4. Determination of DPPH Radical Scavenging Activity

DPPH radical scavenging activity was determined using the DPPH method used by [27], with slight modifications. The stock solution of DPPH was prepared by dissolving 12 mg of DPPH (2,2-diphenyl-1-picrylhydrazyl, MW 394.32 g/mol) in 50 mL of methanol, and was stored in the dark at −20 °C until use. To prepare the DPPH working solution, 10 mL of the stock solution was diluted with 50 mL of methanol (to achieve an absorbance around of 1.5 units at 515 nm), this solution was prepared on the day of the experiment. A volume of 75 µL of the 10-fold diluted extract was mixed with 1.425 µL of DPPH working solution. After that, the mixture was incubated at room temperature and protected from light for 10 min. Water was used as blank. The absorbance was measured at 515 nm using spectrophotometer UV–VIS (UV-1900i, Shimadzu, Kyoto, Japan). The calibration curve (20 to 200 mg L^−1^, with the equation of the line “y = −0.0054x + 1.1481” and R^2^ = 0.9976) was calculated using Trolox as standard, and was conducted in triplicate. Relative (%) of DPPH radical scavenging activity was calculated as the followed Equation (2):

(2) $$Free\;radical\;scavenging\;activity\;\left(\%\right)=\frac{{\mathrm{Abs}}_{control}-Abs_{sample}}{Abs_{control}}\times \;100$$

#### 2.5.5. ABTS Assay

The radical cation decolorization assay (ABTS) was performed according to [28]. A solution of 7 mmol L^−1^ ABTS was mixed with a 2.45 mmol L^−1^ potassium persulfate solution in a 1:1 volume ratio and then incubated at room temperature, protected from light, for 16 h to produce a stock solution of the radical cation ABTS^•+^. After that, a ABTS^•+^ working solution was prepared by diluting 1 mL of the stock solution with 50 mL of ethanol, until reaching an absorbance of 0.700 at 734 nm. For the sample analysis, 30 μL of 10-fold diluted extract was added to a test tube with 3 mL of the ABTS^•+^ radical cation working solution rand allowed to react for 10 min in the dark. The absorbance was measured at 734 nm using spectrophotometer UV–VIS (UV-1900i, Shimadzu, Kyoto, Japan). The calibration curve (20 to 500 mg L^−1^, with equation of the line “y = −0.001x + 0.6988” and R^2^ = 0.9964) was calculated using Trolox as standard, and was conducted in triplicate. Relative (%) of ABTS radical scavenging activity was calculated as the followed Equation (3):

(3) $$Free\;radical\;scavenging\;activity\;\left(\%\right)=\frac{{\mathrm{Abs}}_{control}-Abs_{sample}}{Abs_{control}}\times \;100$$

#### 2.5.6. Oxygen Radical Absorbance Capacity (ORAC) Assay

The ORAC assay was performed according to [29] with adaptations. A stock solution of fluorescein (4 × 10^−5^ mol L^−1^) was previously prepared in a sodium phosphate buffer solution (PBS, 0.075 mol L^−1^ and pH 7.4). From this preparation, a fluorescein working solution (1 × 10^−8^ mol L^−1^) was obtained. AAPH (80 mM) and Trolox calibration solutions were prepared in PBS. For the reaction, 400 µL of the 2-fold diluted extract, Trolox solutions, and PBS solution (blank) were added to a test tube with 2400 µL of fluorescein working solution. After that, the mixture was homogenized and allowed to stand for 10 min in a water bath at 37 °C. Then 1200 µL of AAPH solution was added to each test tube. The fluorescence was measured immediately after adding AAPH and subsequently recorded every 3 min for 30 min. The reaction time was determined based on the blank, when the value dropped to less than 10% of the initial reading. The fluorescence readings were obtained at λ_Excitation_ 480 nm and λ_Emission_ 650 nm, using spectrofluorophotometer (RF-5301PC, Shimadzu, Long Beach, CA, USA). The calibration curve (10 to 400 µM, with equation of the line “y = 0.0091x + 4.6343” and R^2^ = 0.9442) was calculated using Trolox as standard. The concentration of Trolox was plotted on the X-axis against the net area under the fluorescence decay curve (net AUC) on the Y-axis. Trolox and samples AUC was calculated as the followed Equation (4):

(4) $$AUC=1+\frac{FR_{1}}{FR_{0}}+\frac{FR_{x}}{FR_{0}}+\frac{FR_{x}}{FR_{0}}+\cdots +\;\frac{FR_{30}}{FR_{0}}$$

where AUC is area under curve, RFU_0_ is the initial fluorescence reading at 0 min, and RFU_x_ is the fluorescence reading at each subsequent minute.

After determining the AUC for each concentration or sample, it was necessary to calculate the net AUC. This was done using the following formula:

$$Net\;AUC=AUC_{\left(Antioxidant\right)}-\;AUC_{\left(blank\right)}$$

### 2.6. In Vitro Simulated Digestion

In vitro simulated digestion was performed based on the simulated gastric and intestinal phase with slight modifications [30]. Oral digestion was not simulated because it involves a hot beverage, which has only brief contact with the mouth, and the compounds are not digested in this phase [31]. First, 10 mL of each beverage sample (Table 2) were mixed with simulated gastric fluid (2 g L^−1^ NaCl and 3.2 g L^−1^ pepsin, the pH was adjusted to 2.0 ± 0.2 using 6 M and 1 M HCl) respecting the 1:1 (*v*/*v*) ratio. Then, the samples were stirred at 100 rpm at 37 °C for 60 min. Water was used as blank control. Subsequently, the pH of solution was adjusted to 7.0 ± 0.2 using 6 M and 1 M NaOH. After that, the simulated intestinal fluid (10 mM CaCl_2_, 85 mM NaCl, and 1g L^−1^ pancreatin, at pH 7.0 ± 0.2) was mixed with the gastric digestive juice at 1:1 (*v*/*v*) ratio. Then, the samples were stirred at 100 rpm at 37 °C for 120 min. During gastric and intestinal phase, aliquots were collected after 60 and 120 min, respectively. The samples were immediately analyzed to TPC as described in Section 2.5.1.

### 2.7. Statistical Analysis

For statistical analysis of TPC value during in vitro digestion, all of the analyses were carried out in duplicate. Results were reported as mean and evaluated statistically by using one-way analysis of variance (ANOVA). Mean comparisons were performed using Tukey’s post hoc test. Differences were considered significant at *p* < 0.05. All analyses were performed with Minitab 19 Statistical Software.

## 3. Results and Discussion

### 3.1. Central Composite Rotatable Design (CCRD)

**Figure 1:**
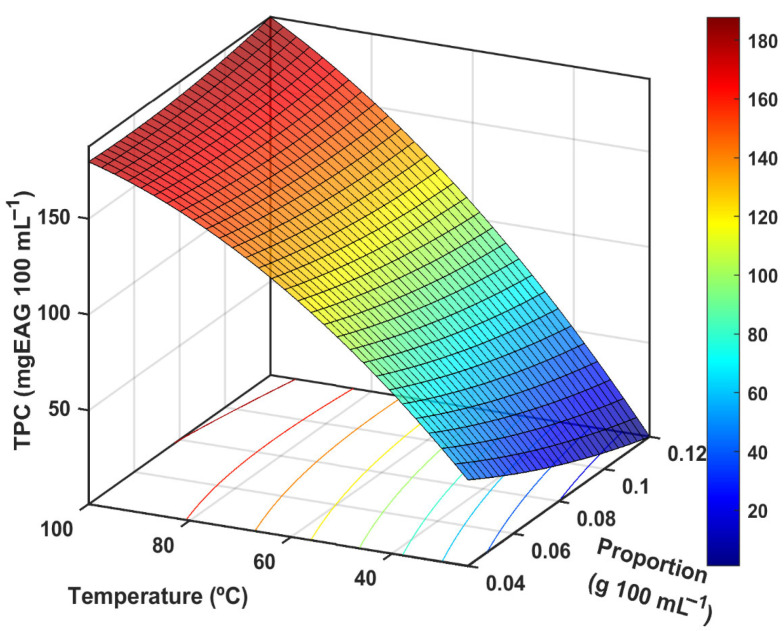
Response surface of roasted açaí seed extract (RASE) proportion (g 100 mL^−1^) and temperature (°C) in total phenolic content (mg gallic acid equivalent 100 mL^−1^).

The CCRD was used to evaluate the interaction of the proportion of roasted açaí seed powder and the extraction temperature of phenolic compounds, including central points in the experimental design. The obtained results were statistically analyzed to describe the relationship between the evaluated factors and TPC. To the best of our knowledge, there are no previous reports of using a mathematical design to optimize the aqueous extraction parameters of TPC in the preparation of the beverage from roasted açaí seed, simulating a homemade process. The experimental results for TPC are presented in Figure 1.

The model showed a significant *p*-value (*p* < 0.05) for the regression coefficient and a non-significant lack of fit (*p* > 0.05). The correlation coefficients (R^2^ and R_adj_^2^) indicated a good fit to the experimental data, demonstrating that the model explained most of the variability in the responses (Table 3). These results suggest that the model can predict the TPC within the evaluated experimental range.

According to Table 4, it is possible to observe that the RASE proportion (b_1_) and the interaction between RASE proportion and temperature (b_1_b_2_) did not show statistical significance (*p* > 0.05). However, temperature (b_2_) and the quadratic term of temperature (b_2_^2^) were the only variables that significantly affected (*p* < 0.05) the total phenolic content (TPC) after extraction.

As the extraction temperature increased from 25 °C to 100 °C, a substantial enhancement in TPC was observed. This effect can be attributed to the improved solubility of phenolic compounds at elevated temperatures, which promotes their diffusion from the plant matrix into the solvent. At lower temperatures, limited molecular mobility within the matrix impairs cell wall disruption and phenolic release, ultimately reducing extraction efficiency [32]. A similar trend was reported in a study employing Response Surface Methodology (RSM) for defatted pitaya seeds, where the interaction between extraction time and temperature significantly affected TPC recovery, with a notable increase observed under shorter extraction times [33]. This supports the hypothesis that high temperatures, combined with the rapid post-extraction filtration used in the present study, contributed to the elevated phenolic content detected at 100 °C.

The absence of a statistically significant effect of RASE proportion (b_1_) on TPC recovery (*p* > 0.05) suggests that, within the tested range, a saturation point may have been reached, beyond which additional solid material does not enhance phenolic extraction. Considering that this infusion is traditionally prepared and consumed under domestic conditions in Brazil, aqueous extraction was conducted at the maximum practical temperature of 100 °C, employing an intermediate proportion of 6.0 ± 0.5 g 100 mL^−1^. This proportion was selected to balance functional, sensory, and economic considerations. Functionally, it provided significant phenolic content with established antioxidant capacity. From a sensory perspective, increasing the RASE concentration could potentially impair the organoleptic properties of the infusion, decreasing consumer acceptability, particularly among populations unfamiliar with the product. Economically, given that RASE-based products are still produced and commercialized at a local scale, maintaining an intermediate concentration ensures a favorable cost–benefit ratio for consumers. This optimized condition yielded a TPC of 21.78 ± 0.77 mg GAE/g and a TFC of 36.23 ± 2.59 mg QE/g, as observed in Table 5. 

The RASE exhibited lower TPC values compared to those reported in previous studies (Table 6). This discrepancy may be attributed to differences in extraction methods and solvents used, which are known to affect the efficiency of polyphenol recovery from açaí seeds. Regarding TFC values, although a direct comparison is limited due to differences in the units of expression, it is worth noting that Previtalli-Silva et al. [34] reported the aqueous fraction of a hydroalcoholic extract as the one with the highest flavonoid recovery (26.60 ± 0.23 μmol QE/g), highlighting the effectiveness of water-based extraction for these compounds.

The chemical composition of fruits, including their TPC and TFC, can be influenced by multiple factors such as climate, location of sample collection, seasonal variations, as well as the extraction methods and solvents employed [37]. Additionally, the present study uniquely focuses on the açaí seed after the roasting process, a condition that may significantly alter the profile and content of bioactive compounds.

These factors collectively may explain the variation in TPC values reported in previous studies, which range from 64.58 to 500 mg GAE/g (Table 6). In the absence of studies specifically addressing roasted açaí seed extracts, comparisons must depend on investigations of unroasted seeds extracted under different conditions. Recent studies have explored açaí seed extracts for applications in the biotechnology, cosmetics, and food industries. For instance, lyophilized açaí seed extracted with an ethanol:water mixture (57:43, *v*/*v*) at 25 °C yielded 64.58 ± 1.89 mg GAE/g [9], while extraction with ethanol:water (1:1, *v*/*v*) at 60 °C resulted in a TPC of approximately 6.69 g GAE/100 g (or 66.90 mg GAE/g-a calculation obtained by the authors) [38].

These comparatively high TPC values can be partially attributed to the use of lower polarity solvents, such as ethanol, which enhance the solubility and diffusion of phenolic compounds [26]. Conversely, Rossetto et al. [1] reported higher TPC values than those obtained in the present study using deionized water. This divergence may be due to their extraction conditions, which involved constant stirring for 37.5 min at 30 °C, facilitating membrane disruption and thereby enhancing the release of phenolic compounds into the aqueous medium. In contrast, the aqueous roasted açaí seed extract (RASE) in the present study was obtained solely through simple filtration, likely minimizing mechanical cell disruption and consequently reducing TPC recovery.

### 3.2. Metabolite Profile by ^1^H NMR

Nuclear Magnetic Resonance (NMR) spectroscopy is a powerful, non-destructive, and untargeted analytical technique widely used for the identification and characterization of metabolites in complex biological samples [39]. However, the application of low-field ^1^H NMR spectroscopy, as conducted in this study, imposes inherent limitations related to its reduced spectral resolution and sensitivity compared to high-field instruments. These limitations complicate the unambiguous identification of metabolites in complex matrices such as plant extracts, where signal overlap and low-intensity peaks are common challenges [40,41]. As a result, the ^1^H NMR analysis performed here provides only preliminary indications regarding the major compounds present in the RASE. Definitive identification and quantification of these metabolites would require more robust and sensitive analytical techniques, such as liquid chromatography-mass spectrometry (LC-MS) or high-performance liquid chromatography (HPLC).

**Figure 2:**
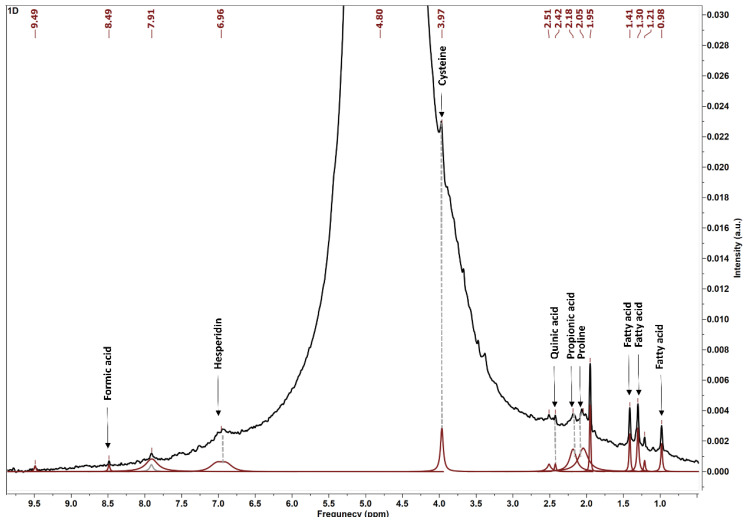
^1^H NMR to visualize the main compounds present in the roasted açaí seed powder extract.

To overcome these challenges and improve the accuracy of metabolite assignments, we employed advanced spectral processing methods, including baseline correction, spectral alignment, and signal deconvolution [42]. The representative spectrum is shown in Figure 2, where the black line corresponds to the total ^1^H NMR spectrum with overlapping peaks, and the red line represents the resolved peaks obtained after applying deconvolution techniques, allowing better resolution and discrimination of individual metabolites. Additionally, metabolite identification was rigorously confirmed through comparison with spectral data from the Human Metabolome Database (HMDB) (https://www.hmdb.ca/) a comprehensive and reliable resource that enhances confidence in compound assignments despite the limitations inherent to low-field NMR [43].

Using this approach, a detailed qualitative analysis of the metabolite profile of RASE was performed. The principal metabolites identified in the aqueous extract were classified into fatty acids, amino acids, organic acids, and phenolic compounds. Intense resonances observed in the *δ* 0.8–3.0 ppm region correspond to methyl and methylene groups from acyl chains, predominantly derived from fatty acids [44]. Amino acids such as cysteine and proline were also detected, complementing the biochemical profile of the extract. The spectral region between δ 1.95 and 2.51 ppm revealed signals attributable to chlorogenic acids (CGAs), while organic acids including propionic, quinic, and formic acids were identified, with formic acid showing a characteristic resonance near δ 8.49 ppm corresponding to its non-carboxylic proton, typically observed within the δ 8.2–8.5 ppm range depending on pH [45]. These assignments are summarized in Table 7, which details the chemical shifts and corresponding metabolites identified.

Notably, the aromatic region (*δ* 5.7–9.5 ppm) exhibited only weak signals around δ 6.96 ppm, indicating a relatively low abundance of phenolic compounds such as hesperidin metabolites. These signals align with acidic protons of phenolic compounds (near δ 8.5 ppm) and aromatic protons of polyphenols (*δ* 6.0–7.5 ppm) [50]. This finding is consistent with previous studies reporting açaí seeds as rich sources of amino acids and lipids [48], a conclusion corroborated here by the substantial presence of resonances corresponding to these biomolecules. Although phenolic compounds showed low signal intensity, the antioxidant activity observed in RASE can be largely attributed to their amino acid content. In particular, cysteine (*δ* 3.97 ppm) is recognized for its strong antioxidant capacity due to the presence of its thiol group (-SH), which acts as an effective scavenger of reactive oxygen species. In addition, other amino acids, such as proline, can contribute synergistically to the antioxidant potential, reinforcing the overall bioactivity of the extract [51].

Organic acids are of particular interest given their influence on the sensory characteristics of the extract and derived beverages. Similar metabolites have been described in roasted coffee, where organic acid concentrations, especially in lightly to moderately roasted beans, contribute to acidity and fruity flavor attributes [52]. Chlorogenic acids, a group of phenolic acids, also contribute to the acidity and bitterness of coffee [53]. Higher roasting temperatures promote the degradation of CGAs into quinic acid, which enhances bitterness and astringency [54].

Although studies employing ^1^H NMR for açaí pulp and seed characterization are scarce, previous reports describe flavonoid compounds such as orientin, homoorientin, vitexin, luteolin, chrysoeriol, and quercetin in solvent fractions of açaí [55]. Thermal processing like roasting, as applied to açaí seeds, may reduce the levels of phenolic compounds detectable by ^1^H NMR, similar to oxidative processes reported in black tea compared to green tea, which promote enzymatic oxidation and compound condensation [56]. Despite possible reductions, the observed spectral peaks in RASE confirm the presence of various bioactive molecules, including fatty acids, amino acids, and organic acids. These compounds likely contribute to the antioxidant and anti-inflammatory effects commonly associated with açaí, underscoring its potential as a rich source of bioactive constituents [57].

### 3.3. Phytochemical Profile of the Roasted Açaí Seed Extract

This study evaluated TPC, TFC and antioxidant capacity by four methods using an extract with a proportion of 6.0 ± 0.5 g 100 mL^−1^ (intermediate value between the extremes used in the present study) and temperature of 100 °C, as can be seen in Table 5.

#### Antioxidant Capacity

To comprehensively assess the antioxidant potential of the extract, it is essential to employ complementary assay methods [58]. Table 5 indicates the antioxidant capacity of roasted açaí seed extract beverage by four complementary methods which were used based on ferric-reducing antioxidant power (FRAP), free radical scavenging assay (DPPH and ABTS), and oxygen radical absorbance capacity (ORAC). The antioxidant capacity of roasted açaí seed extract using FRAP is represented by a 183.33 µM TE/g; and DPPH represented by a 23.06 mg TE/g and free radical scavenging activity 66.29%. By the ABTS method, 51.63 mg TE/g and free radical scavenging activity 45.21%. ORAC antioxidant assay delivered 31.46 mol TE/g in the extract.

Regarding the methods selected to determine antioxidant activity, FRAP method relies on the reduction power of antioxidants present in the extract, specifically through the reduction of the Fe^3+^-TPTZ complex to the Fe^2+^-tripyridyltriazine complex. This reduction is facilitated by electron-donating antioxidants under low pH conditions [26]. DPPH, ABTS and ORAC are methodologies widely used in food research due to their relevant antioxidant mechanism [59]. DPPH is measured by the reduction of purple stable free radical DPPH to the yellow hydrazine carried out by capturing the unpaired electron and the extent of discoloration indicating the samples’ scavenging activity [13]. Regarding ABTS, the antioxidant capacity is measured by this method through the quenching of ABTS^•+^ radicals via an electron transfer reaction, which can eliminate the generated radical [57]. Finally, ORAC assay measures the antioxidant activity that most closely resembles physiological conditions [12].

The presence of TPC in the RASE underscore the importance of evaluating its antioxidant activity across different methods. Previous studies have shown that these compounds can inhibit free radicals and oxygen-stimulated reactions, thereby protecting biological systems from oxidative stress and inflammatory responses [60,61,62]. Free radicals are known to contribute to a range of harmful health effects. The present results indicate that RASE exhibits significant antioxidant potential across different assay methods, emphasizing its relevance to human health and its potential role in managing diseases linked to oxidative stress [26]. A diet rich in antioxidants may reduce cellular and DNA damage, potentially slowing the progression of various diseases, including neurodegenerative disorders such as Alzheimer’s and Parkinson’s disease [63,64].

Previous studies reported higher antioxidant activity of açaí seed extract (mainly from unroasted seeds), obtained using different solvents (polar and less polar) and extraction methods (e.g., ultrasonic bath). Melo et al. [9], observed strong in vitro antioxidant activity by DPPH (622.81 ± 67.56 µmol/g) and ABTS (763.09 ± 17.27 µmol TEAC/g), in a lyophilized açaí seed extract with ~60% ethanol. Soares et al. [65] reported higher antioxidant activity by FRAP (582.8 ± 15.0 mmol Fe^2+^/100 g) and TEAC (642.4 ± 13.1 mmol Trolox/100 g) using ethanol:water solvent. Moreover, methanolic açaí seed extract showed 3835.44 ± 73.50 μmol Trolox/g in TEAC measurements and 4082.16 ± 58.55 μmol. TE/g by ORAC assay [12]. Meanwhile, a lyophilized aqueous extract exhibited EC_50_ 8.8 ± 0.2 in DPPH scavenging [13]. Expanding the comparison to studies that evaluated the antioxidant activity of other beverages with the same proposal of a coffee substitute, it was observed by Komes et al. [66] that of analyzed 10 commercially offered beverages, the beverage produced with 100% roasted chicory presented better antioxidant activity by ABTS (8.19 ± 0.03 mmol Trolox L^−1^) and DPPH (4.39 ± 0.03 mmol Trolox L^−1^), while beverages formulated with 100% roasted barley presented lower values for antioxidant capacity by ABTS (1.11 ± 0.02 mmol Trolox L^−1^) and DPPH (0.61 ± 0.00 mmol Trolox L^−1^). From the same perspective, tamarind seed under different roasts (light, medium and dark), obtained by aqueous extraction, demonstrated an antioxidant activity by the DPPH assay of 94.17% to 73.47% [67]. It is important to highlight the difference between our results and previous studies could be explained by variations solvents, extraction techniques, location, and season can influence on total phenolic content and that impact on antioxidant activity [68].

### 3.4. In Vitro Simulated Digestion

Following the identification of the antioxidant activity of isolated RASE, this study aimed to evaluate the interaction of its metabolites with a food matrix, such as milk, which is commonly consumed with coffee in Brazil, and to assess how this combination behaves under simulated gastrointestinal digestion conditions. As shown in Table 8, before in vitro simulated digestion, the combination of RASE with whole cow milk and whole goat milk resulted in the highest concentrations of TPC, reaching 47.42 and 54.31 mg GAE 100 mL^−1^, respectively. The TPC values of RASE combined with whole milk (both cow and goat) were significantly higher (*p* < 0.05) than those observed for RASE combined with semi-skimmed or skimmed milk, as well as RASE alone. This pattern is consistent with previous findings, which show that both goat and cow milk naturally contain phenolic compounds. Specifically, goat milk with 4.07 ± 1.47% fat showed a TPC recovery of 69.03 ± 6.23 mg GAE L^−1^, while cow milk with 3.76 ± 0.83% fat showed a TPC recovery of 49.00 ± 10.77 mg GAE L^−1^, with no significant difference between them (*p* > 0.05) [69], which corroborates the trend observed in the present study. Similarly, Quan et al. [70] reported that the combination of coffee with whole cow milk resulted in a higher bioaccessibility of phenolic compounds compared to coffee alone or coffee combined with skimmed cow milk. Furthermore, the addition of milk to tea and cocoa infusions has also been shown to enhance the phenolic content [71]. These findings suggest that the chemical composition of milk, particularly its fat content, influences the recovery and potential bioaccessibility of phenolic compounds. A positive correlation between milk fat and TPC in coffee-milk systems has been established, likely due to the interaction between phenolic compounds and fat molecules, which may facilitate their incorporation into fat globules [72].

After 60 min of the gastric phase (GP), a reduction in TPC values was observed, indicating that pH and enzymatic conditions alter the content of bioactive compounds. In this phase, the combination of RASE with whole goat milk exhibited the highest TPC value, whereas RASE alone presented the lowest TPC (*p* < 0.05). Notably, the interaction of RASE with semi-skimmed and skimmed cow milk resulted in similar TPC levels, reflecting the same trend observed in the pre-digestion phase. The acidic environment of this phase (pH 2.0 ± 0.2) may contribute to the instability of larger phenolic molecules. Additionally, it has been proposed that pH variations during simulated digestion affect the distribution of hydroxyl radicals (-OH) on the rings of phenolic compounds [72,73].

After 120 min of the intestinal phase (IP), a further decrease in TPC was also observed. At this stage, the combination with whole goat milk maintained the highest TPC value, which was significantly different from RASE alone (*p* < 0.05). Whole goat milk demonstrated notable levels of phenolic compounds after the gastrointestinal digestion (*p* < 0.05). To date, no previous studies have evaluated the interaction between whole goat milk and coffee, tea, or extracts under these same conditions. However, it has been suggested that the Folin–Ciocalteu reactivity of the extract with whole goat milk may be influenced by the presence of non-phenolic compounds such as free amino acids, peptides, and proteins [74,75,76], which could affect the observed TPC values.

The size of casein micelles varies between species, ranging from 220–270 nm in goat milk to 142–229 nm in cow milk [77]. Although the internal structure of caseins from different species shows similarities, goat milk casein contains lower levels of αs1-casein compared to cow milk, while β-casein is more abundant [78]. During in vitro digestion, this structure may form a soft or loose curd under acidic pH conditions (as in the simulated stomach), facilitating easier digestion compared to the firmer curd formed by cow milk [79]. A lower proportion of undigested casein has been reported in whole goat milk compared to whole cow milk during the first 60 min of static in vitro gastric digestion at pH 3.0. This is likely due to the more fragile nature of the curd formed by goat milk [80]. Consequently, the hydrolysis of goat milk proteins occurs more rapidly and extensively than that of cow milk proteins, leading to an increased presence of peptides and amino acids [81,82], which are detectable by the Folin–Ciocalteu method, as previously mentioned.

In contrast to our findings, an increase in the release of phenolic compounds from rose tea combined with skimmed cow’s milk at 25 °C has been reported. The authors observed that, after gastric digestion, there was an increase in total phenolic content, chlorogenic acid, and quercetin by 10.61%, 4.01%, and 7.02%, respectively, compared to rose tea at 25 °C. Furthermore, after intestinal digestion, the interaction of rose tea with milk promoted an increase in total phenolic content and chlorogenic acid by 14.17% and 12.56%, respectively [83]. However, other studies have reported a similar tendency for TPC decrease during gastrointestinal digestion. In the present study, all treatments showed a 65–76% reduction in TPC after the intestinal phase compared to the pre-digestion phase. A comparable reduction was observed in a study evaluating Terebinth (*Pistacia terebinthus* L.) coffee combined with milk (whole and skimmed), showing a 12–71% lower TPC compared with undigested samples after gastrointestinal digestion [84]. Similarly, roasted yerba mate (*Ilex paraguariensis*) extract combined with cow milk (whole, semi-skimmed, and skimmed) showed an average TPC reduction of 55% after gastric digestion. Interestingly, an increase in TPC of 3–6% was observed in the intestinal phase for extracts obtained with whole and semi-skimmed milk, whereas the infusion made with skimmed milk showed a 28% increase [31].

During each stage of simulated in vitro digestion, a decrease in TPC was observed, which can be explained by the susceptibility of phenolic compounds to oxidative degradation, transformation, polymerization, and complexation with proteins [83]. Nonetheless, whole milk demonstrated the best capacity to preserve phenolic content after the intestinal phase. This suggests that the interaction of lipids with phenolic compounds protects them during gastrointestinal digestion [22,70,85]. It is important to emphasize that the effect of phenolic compounds on human health depends not only on their quantity in foods but also on factors such as stability, microbiota interaction, and digestive enzymes. The size of phenolic compounds influences their absorption site in the human body; smaller molecules are more readily absorbed in the small intestine, while more complex molecules are primarily absorbed in the large intestine. Regardless of the absorption site, these molecules undergo conversion into low molecular weight metabolites via methylation, sulfation, and glucuronidation reactions [86]. Furthermore, the biotransformation mechanisms induced by intestinal microbiota affect the bioavailability of these phenolic compounds. Their absorption and metabolism influence their biological actions in the body, which may include antioxidant, anti-inflammatory, immunomodulatory, and anti-tumor effects [87,88,89].

The in vitro digestion model revealed a significant reduction in the total phenolic content (TPC) across all treatments, as evidenced by the high TPC reduction percentages, ranging from 65% to 76%. Notably, RASE combined with whole goat milk exhibited the highest TPC in the intestinal phase (17.48 ± 1.03 mg GAE 100 mL^−1^). Conversely, RASE alone presented one of the lowest bioaccessibility rates (24% ± 4), similar to the combination with whole cow milk (24% ± 1), both with the highest TPC reduction (76% ± 4). Interestingly, samples combined with semi-skimmed and skimmed milk showed moderately higher bioaccessibility (33–35%) and correspondingly lower TPC reductions (65–67%). These findings suggest that the fat content of milk may play a role in modulating the stability and release of phenolic compounds during gastrointestinal digestion. Previous studies have indicated that interactions between phenolic compounds and milk proteins or lipids can influence their bioaccessibility by forming complexes that hinder or facilitate their release in the intestinal phase [90,91].

Moreover, the observed inverse relationship between initial TPC and post-digestion bioaccessibility highlights the complexity of phenolic compound behavior in food matrices. Despite the higher phenolic concentrations before to digestion in whole milk systems, these compounds may be more susceptible to degradation or entrapment during digestion. These results align with reports indicating that phenolic compounds in complex matrices often undergo significant structural transformations, impacting their stability and subsequent bioaccessibility [31,92]. Overall, the antioxidant activity initially observed in RASE is closely associated with key metabolites identified by ^1^H NMR, particularly phenolic compounds. The in vitro digestion results showed that these bioactive compounds undergo structural changes throughout the digestive process, influencing both their concentration and antioxidant capacity. This clear link between chemical composition, initial antioxidant potential, and post-digestion bioactivity highlights the functional relevance of RASE and reinforces its potential as a phenolic-rich ingredient for the development of functional beverages.

## 4. Conclusions

This study highlights the potential of roasted açaí seed (a by-product of the açaí industry) as a novel source of bioactive compounds with antioxidant properties. Notably, the combination of RASE with semi-skimmed and skimmed bovine milk enhanced the bioaccessibility of phenolic compounds following the intestinal phase of in vitro digestion. These results suggest that RASE-based formulations could be further explored as functional beverages or natural antioxidant supplements. Future research should focus on the identification and quantification of all compounds present in the beverage, as well as the evaluation of their bioavailability and the investigation of their metabolic effects in both cellular and in vivo models. Additionally, exploring their antimicrobial activity against pathogenic bacteria may reveal further functional applications. Overall, this study underscore the importance of understanding food matrix–digestion interactions as a strategy to enhance the functionality of phenolic-rich ingredients. Moreover, it supports the valorization of agro-industrial by-products, contributing to both environmental sustainability and the development of health-promoting food products.

## Figures and Tables

**Table 1:** Levels and real values for the Central Composite Rotatable Design (CCRD).

	Level		Real Values	
Run	×1	×2	×1 (g 100 mL^−1^ H_2_O)	×2 (°C)
**1**	−1	−1	5.2	36
**2**	−1	1	5.2	89
**3**	1	−1	10.8	36
**4**	1	1	10.8	89
**5**	−1.41	0	4	63
**6**	1.41	0	12	63
**7**	0	−1.41	8	25
**8**	0	1.41	8	100
**9**	0	0	8	63
**10**	0	0	8	63
**11**	0	0	8	63

**Table 2:** Roasted açaí seed extract and the interaction of extract plus cow milk (whole, semi-skimmed, and skimmed) and goat milk (whole) made during in vitro simulated digestion.

Treatment	Mixing Ratio
RASE	-
RASE + whole milk	1:1 (*v:v*)
RASE + semi-skimmed milk	1:1 (*v:v*)
RASE + skimmed milk	1:1 (*v:v*)
RASE + whole goat milk	1:1 (*v:v*)

RASE: roasted açaí seed extract; -: not mixing ratio was used.

**Table 3:** ANOVA for the models describing total phenolic content (TPC).

Model		Sum of Square	DF	Mean Square	*p*-Value
**TPC** R^2^ = 0.9884 R_adj_^2^ = 0.9769	Regression	2.6842 × 10^4^	5	5.3684 × 10^3^	7.7217 × 10^−5^
	Error	826.9629	3	275.6543	0.0726
	Lack of fit	142.9250	3	47.6417	0.6931
	Pure error	171.2065	2	85.6033	
	Total SS	2.7156 × 10^4^	10	2.7156 × 10^3^	

**Table 4:** Regression coefficients of the fitted model for total phenolic content (TPC).

Coefficients	TPC (mg GAE 100 mL^−1^)	*p*-Value
b_0_	9.3285	0.8385
b_1_	−1.1400	0.1894
b_2_	3.1365	0.0080
b_1_ b_2_	8.8283	0.1556
b_1_^2^	2.2336	0.6151
b_2_^2^	−0.0137	0.0347

TPC: total phenolic content; b_0_: intercept; b_1_: proportion (g 100 mL^−1^); b_2_: temperature (°C); b_1_b_2:_ interaction between proportion and temperature; b_1_^2^ and b_2_^2^: quadratic terms for proportion and temperature, respectively.

**Table 5:** Phytochemical screening and antioxidant capacity of roasted açaí (*E. oleracea*) seed extract.

**Roasted Açaí Seed Extract**	**Phytochemical Screening**		**Antioxidant Capacity**					
	**TPC**	**TFC**	**FRAP**	**DPPH**		**ABTS**		**ORAC**
	**mg GAE/g**	**mg QE/g**	**µM TE/g**	**mg TE/g**	**Free Radical Scavenging Activity (%)**	**mg TE/g**	**Free Radical Scavenging Activity (%)**	**mol TE/g**
	21.78 ± 0.77	36.23 ± 2.59	183.33 ± 9.71	23.06 ± 0.72	66.29 ± 2.23	51.63 ± 2.35	45.21 ± 0.07	31.46 ± 1.60

TPC: total phenolic content; TFC: total flavonoid content; QE: quercetin equivalent; GAE: gallic acid equivalent; TE: Trolox equivalent. Data are the means ± SD (two replicates).

**Table 6:** Studies that recovered, quantified, and identified total phenolic content in açaí seeds extract under different methods and types of solvents.

Açaí Seed Solvent Extraction	Extraction Method/Conditions	Analytical Method	Polyphenol Concentration	Ref.
Ethanol:water (57:43, *v:v*)	At room temperature (25 °C)	Folin-Ciocalteau	64.58 ± 1.89 mg GAE/g	[9]
-	Supercritical Anti-Solvent	Folin-Ciocalteau	261 ± 3 mg GAE/g	[35]
Ethanol	Supercritical Anti-Solvent	Folin-Ciocalteau	500 ± 5 mg GAE/g	[35]
Ethanol:water (1:1, *v:v*)	Solid/liquid ratio of 1:8 (*m:v*), for 60 min at 50 °C under orbital agitation at 120 rpm	RP-HPLC-DAD	Peak at 51 min (polymeric procyandins)	[12]
Deionized water	Solid/liquid ratio of 1:30 (*m:v*), for 37.5 min at 30 °C at 75 rpm	Folin-Ciocalteau	1805 to 1893 mg GAE L^−1^	[1]
Distilled and deionized water	Macerated for 30 min at 25 °C	HPLC-MS-DAD	Total proanthocyanidins (158 ± 1 mg EC/g)	[13]
Hydroalcoholic	Macerated for 2 h, shaking and kept at 4 °C for 10 days	Folin-Ciocalteau	28.3%	[36]

-: not used solvent; GAE: gallic acid equivalent; EC: equivalents of catechin; RP-HPLC-DAD: reversed-phase high-performance liquid chromatography; HPLC-MS-DAD: high-performance liquid chromatography coupled with diode-array detection and electrospray ionization tandem mass spectrometry.

**Table 7:** Metabolites present in ^1^H NMR spectra of roasted açaí seed extract, with chemical shift in ppm.

Metabolite	^1^H Chemical Shifts δ, ppm	Ref.
**Lipids**		
Fatty acids	0.98, 1.30, 1.41	[46]
**Amino acids**		
Proline	2.05	[47]
Cysteine	3.97	[48]
**Organic acids**		
Propionic acid	2.18	[39]
Quinic acid	2.42	[48]
Formic acid	8.49	[49]
**Phenolics**		
Hesperidin	6.96	[48]

**Table 8:** Total phenolic compounds (TPC) of roasted açaí seed extract with cow and goat milk at each in vitro simulated digestion.

	Total Phenolic Compounds (mg GAE 100 mL^−1^)			Bioaccessibility (%) *	TPC Reduction (%) †
Treatment	Before Digestion (BD)	Gastric Phase (GP) After 60 min	Intestinal Phase (IP) After 120 min		
**RASE**	35.89 ± 1.95 ^B^	19.40 ± 2.23 ^C^	8.67 ± 1.53 ^C^	24% ± 4 ^B^	76% ± 4 ^A^
**RASE + whole cow milk**	47.42 ± 0.45 ^A^	29.30 ± 2.68 ^B^	11.21 ± 0.48 ^B^	24% ± 1 ^B^	76% ± 1 ^A^
**RASE + semi-skimmed cow milk**	33.72 ± 2.30 ^B^	23.33 ± 0.50 ^BC^	11.13 ± 1.06 ^B^	33% ± 5 ^A^	67% ± 5 ^B^
**RASE + skimmed cow milk**	31.74 ± 3.31 ^B^	24.67 ± 1.30 ^BC^	10.90 ± 1.00 ^BC^	35% ± 4 ^A^	65% ± 4 ^B^
**RASE + whole goat milk**	54.31 ± 0.93 ^A^	43.06 ± 3.49 ^A^	17.48 ± 1.03 ^A^	32% ± 5 ^AB^	68% ± 5 ^AB^

*: bioaccessibility after the intestinal phase; †: TPC reduction after the intestinal phase; RASE: roasted açaí seed extract; BD: before digestion; GP: gastric phase; IP: intestinal phase. Averages that do not share a letter in the same column are significantly different (*p* < 0.05). Results are expressed in mg GAE 100 mL^−1^. Data are the means ± SD (two replicates).

## Data Availability

The original contributions presented in this study are included in this article. Further inquiries can be directed to the corresponding author.
